# Current Advances in Red Blood Cell Generation Using Stem Cells from Diverse Sources

**DOI:** 10.1155/2019/9281329

**Published:** 2019-07-29

**Authors:** Yoojin Seo, Kyung-Hwa Shin, Hyung Hoi Kim, Hyung-Sik Kim

**Affiliations:** ^1^Institute of Translational Dental Sciences, School of Dentistry, Pusan National University, Yangsan 50612, Republic of Korea; ^2^Department of Life Science in Dentistry, School of Dentistry, Pusan National University, Yangsan 50612, Republic of Korea; ^3^Department of Laboratory Medicine, Biomedical Research Institute, Pusan National University Hospital, Pusan National University School of Medicine, Busan, Republic of Korea

## Abstract

Blood transfusions hold an indispensable part in the modern healthcare system. Up to date, the blood supply is largely dependent on donations. Unfortunately, collecting the clinical-grade blood products has become a challenging mission due to accelerated population aging, which not only increases the need for blood transfusions but also decreases the number of healthy donors. Moreover, individuals with severe hematological abnormalities or rare blood phenotypes need alternative therapeutic approaches instead of conventional blood transfusion. In these aspects, the concept of *in vitro*/*ex vivo* production of blood cells has been emerging and many attempts have been focused on manufacturing mature erythrocytes, so-called red blood cells (RBCs), the most common and important component among the blood derivatives. In this review, we provide a general overview regarding the current strategies for generating RBCs from various stem cell sources including pluripotent stem cells (PSCs) as well as circulating blood stem cells and the remaining challenges that must be overcome prior to their practical application.

## 1. Introduction

The clinical demand for blood transfusion remains high in surgical interventions and hematologic malignancies. However, the imbalance in blood supply and demand has been intensified due to demographic aging, increasing outbreaks in the transmission of infectious diseases such as Ebola and dengue, and limited compatibility of donor blood [1, 2]. The aging of the population reduces the number of healthy donors and increases the incidence of diseases that require transfusion [3]. Also, transfusion-transmitted blood-borne disease is not completely controlled by present technology, causing safety issues [4]. Another major problem with the conventional blood supply system is the lack of blood products for patients with multiple alloantibodies or high incidence antigens as well as rare blood types [5]. Therefore, *in vitro* generation of universal blood substitutes has been widely investigated in an attempt to alleviate clinical dependence on blood donation and to resolve unmet clinical needs [6, 7].

Erythropoiesis is a developmental procedure in which multipotent hematopoietic stem/progenitor cells (HSPCs) become restricted to generate circulating RBCs [8]. Upon cell fate commitment to the erythroid lineage, HSPCs lose their self-renewal potential and begin to differentiate into erythroid progenitors that consist of active-dividing erythroid burst-forming units (BFU-E) followed by less-proliferative erythroid colony-forming units (CFU-E). After further maturation, serial intermediate stages of erythroblasts referred to as proerythroblasts and basophilic, polychromatophilic, and orthochromatic erythroblasts give rise to reticulocytes which then terminally differentiated into mature RBCs. The entire process occurs within the bone marrow niche composed of both cellular and extracellular interactions and is regulated by several bioactive molecules such as growth factors, cytokines, and hormones [9, 10]; therefore, making functional RBCs *in vitro* is still a challenging mission.

Since fully differentiated RBCs are not proliferative, the establishment of expandable HSPCs and/or erythroid progenitors amenable to an erythropoiesis-like maturation process *in vitro* has been the priority issue for the RBC production. In past decades, researchers have successfully generated RBCs in the laboratory and proved their therapeutic potentials with animal models [11]. Several strategies have been devised to obtain RBCs *in vitro*: (1) recapitulating the developmental hematopoiesis towards erythrogenesis using pluripotent stem cells (PSCs), (2) reprogramming the fate of either stem cells or somatic cells to hematopoietic lineage, and (3) stimulating the expansion and maturation of circulating HSPCs isolated from the umbilical cord blood (UCB) and peripheral blood (PB) [12, 13]. Since the general basis of RBC production has been established, researchers are now trying to optimize the procedure to obtain a sufficient number of functional RBCs in the most convenient and economical way (Table 1). For instance, immortalization of HSPCs or erythroid progenitors has been tried recently to obtain a sufficient number of mature RBCs. In this review, we introduce current RBC generation strategies with the latest advances and discuss remaining challenges for the clinical application to provide an overview of the current status of this field.

## 2. Recapitulating Developmental Hematopoiesis with PSCs

Thanks to the monumental work by Yamanaka and his colleagues, induced PSCs (iPSCs) can be obtained from a wide range of somatic cells using highly reproducible, well-defined protocols [14, 15]. This breakthrough enables us to utilize pluripotent stem cells for therapeutic purposes with less-ethical concerns compared to embryonic stem cells (ESCs) [16, 17]. Similar to ESCs, iPSCs are immortal cells with the infinite self-renewal capability and they can generate embryonic bodies (EBs) which spontaneously differentiate into three germ layers—ectoderm, mesoderm, and endoderm *in vitro* and *in vivo*. Since EB formation recapitulates several key aspects of embryonic development, it is possible to bias the direction of differentiation towards intended lineage or cell type through modulation of transcriptional factor activity and morphogenic signals, as observed *in vivo* [18]. Therefore, many attempts have been conducted to transform PSCs into RBCs via sequential modification of culture conditions after EB formation [19]. In general, the differentiation procedure consists of two stages: step 1, the generation of HSPCs derived from PSCs, and step 2, lineage specification of HSPCs into mature RBCs.

Up to date, several bioactive components including interleukin- (IL-) 3, IL-6, Flt3 ligand (Flt3-L), granulocyte colony-stimulating factor (G-CSF), stem cell factor (SCF), and thrombopoietin (TPO) are suggested as the essential factors during embryonic hematopoiesis [20–23]. In addition, activin A is known to stimulate the commitment of the mesodermal lineage into hematopoietic fate via the activin/nodal signaling pathway [24], while bone morphogenetic protein-4 (BMP-4), a critical morphogen responsible for the dorsal-ventral axis orientation during the early embryo stage, could increase the CD34^+^ hematopoietic population with high self-renewal property [24, 25]. These well-defined chemical combinations are widely used to promote the hematopoietic differentiation of EBs, and additional treatment with erythroid-promoting factors such as erythropoietin (EPO) [26] and vascular endothelial growth factor (VEGF) [27] can stimulate the further differentiation of PSC-derived HSPCs into RBCs [28, 29].

In addition to the EB method, stromal feeder cells can support the hematopoietic commitment of PSCs as a major cellular component during hematopoietic development *in vivo*. Choi et al. have found that the immortalized mouse bone marrow-derived stromal cell OP9 could stimulate the hematopoietic erythromyeloid differentiation of iPSCs [30, 31] as observed in ESCs [32, 33]. Stromal cells derived from the murine embryonic aorta-gonad-mesonephros region have been reported to possess a high capacity for supporting HSPC generation and expansion *in vitro* [34]. Meanwhile, several groups have isolated HSPCs from iPSC-derived teratoma tissue after *in vivo* transplantation. Teratoma-derived HSPCs possess multipotent differentiation potentials that can successfully reconstitute the hematopoietic system of immunocompromised recipient mice, suggesting that teratoma may act as a hematopoietic niche [35, 36]. Although this approach cannot be applied to the practical field due to safety concerns, it can contribute to disease modeling and genetic studies for hematologic diseases in the preclinical settings.

In 2007, Hanna et al. generated iPSC-derived HSPCs and proved their therapeutic potential for an autosomal recessive hematological disorder with abnormal hemoglobin-possessing RBCs, sickle cell anemia (SCA) [37]. Briefly, they generated autologous mouse iPSCs using a dermal fibroblast isolated from a humanized knock-in SCA model and conducted gene correction to establish normal iPSC lines. iPSCs were then forced to aggregate to form EB under hematopoietic conditions to stimulate the hematopoietic differentiation. Finally, obtained iPSC-derived HSPCs were transplanted into donor mice and therapeutic outcomes were evaluated. They revealed that iPSC-derived blood cells were successfully engrafted and pathological characteristics of RBC defects found in SCA were improved in cell-treated mice, implying that iPSC-derived HSPCs might be fully differentiated into normal RBCs and replace the impaired blood system *in vivo*. Similarly, Raya et al. collected adult fibroblasts from the patient with Fanconi anemia (FA), a rare genetic disorder characterized by hematological abnormalities due to bone marrow failure [38]. They corrected mutated genes to remedy the genetic defect then converted them into iPSCs (FA-iPSCs) followed by EB formation in the presence of OP9 to induce efficient hematopoietic differentiation. It was noted that gene-corrected FA-iPSCs gave rise to the normal hematopoietic lineages as FA-free iPSCs after *in vivo* implantation in mice, emphasizing the therapeutic potential of patient-specific iPSC-derived HSPCs after gene correction for genetic hematological disorders.

In addition, immortalization of PSC-derived erythroid progenitors via genetic modification would contribute to scale up the *ex vivo* RBC production. Takayama et al. has introduced an immortalization strategy for the megakaryocyte-erythrocyte progenitor generation from iPSCs using the transient c-Myc expression system during the HSPC induction [39]. They modified the original hematopoietic differentiation protocol to enhance erythroid specification and found that the presence of EPO in culture media could increase the proliferation as well as differentiation of c-Myc overexpressed iPSCs towards the erythroid lineage, although these differentiated cells seemed to undergo apoptosis [40]. To prevent apoptotic cell death, they additionally transduced PSCs with a Dox-inducible expression vector for the B-cell lymphoma-extra large (Bcl-xL) gene, a member of the Bcl-2 family which functions as an antiapoptotic protein. Of note, overexpression of both c-Myc and Bcl-xL enables erythroblastic cells to expand exponentially. Finally, they could establish immortalized erythrocyte progenitor cell lines using clonal selection and confirmed their maturation capacity into RBCs and reconstitution ability *in vivo*. In another study, Kurita et al. have generated immortal erythroid progenitors from iPSCs using a 2-step protocol [41]; briefly, they overexpressed one hematopoietic transcription factor Tal1 in iPSCs to induce erythroid differentiation more effectively then cultured cells with hematopoietic culture condition for 16 days. Next, differentiated HSPCs were immortalized by transformation with E6/E7 proteins. These established cell lines could proliferate continuously and maintain the cell division rate for more than a year. They also reported that gradual hemoglobin production with an increase in the mature RBC marker expression such as glycophorin (GPA) was observed in these cells upon erythroblastic differentiation, demonstrating the practical potential of immortalized HSPC and/or RBC lines derived from PSCs as a replacement for conventional blood products.

## 3. Cell Fate Reprogramming Using Hematopoietic Transcription Factors (TFs)

The main concept of iPSC generation is to convert fully differentiated cells into ESC-like primitive pluripotent stem cells. With defined four TFs, Oct3/4, Sox2, Klf4, and c-Myc, it is possible to reverse the developmental process and reoriented somatic cell fate to iPSCs [14, 15]. As described above, iPSCs then undergo “directed differentiation” into the target lineage using specific growth factors and morphogens; however, this process requires a comprehensive understanding in developmental biology to mimic the optimal *in vivo* differentiation condition as well as a time- and cost-consuming *in vitro* culture procedure.

In this respect, the advanced technique so-called “direct conversion” has been evolved. Based on the genome-wide expression profiling data, researchers have analyzed the global transcriptome patterns during the lineage commitment and discovered several cell type-specific master TFs that initiate and/or govern the differentiation procedure. Importantly, when these TFs are introduced to somatic cells, they reprogram the cell fate directly to the intended lineage without going through a pluripotent state. Using this technique, several cell types including neural stem cells [42], hepatocytes [43], cardiomyocytes [44], and pancreatic cells [45] can be successfully transdifferentiated from somatic cells.

In the past few years, multiple studies have reported the various combinations of TFs to achieve hematopoietic conversion [10, 46]. Up to date, the most frequently used reprogramming factor is hematopoiesis-governing TF Gata2, and other related TFs such as Lmo2, Tal1, Scl, Fos, Gfi1B, and Erg should be combined to reprogram the fate of somatic cells as such into the hematopoietic lineage [47–49]. Most of these TFs are known to correlate with the hematopoietic specification during the embryonic development. After hematopoietic TF overexpression, somatic cells are forced to undergo de-differentiation into the hemogenic endothelium stage then acquire HSPC-like characteristics with multilineage differentiation potentials. Interestingly, the ectopic expression of single factor Oct4, which is the essential TF for the acquisition of pluripotency, could transform fibroblasts to the HSPC-like population in the hematopoietic culture condition; however, the derivative cells exhibited limited multipotency as well as poor engraftment capacity [50]. Mitchell et al. also applied this method to reprogram the fibroblast fate and found that the Oct4-expressing fibroblast could generate either hematopoietic cells or neuronal progenitors depending on the composition of media utilized in the reprogramming process [51]. This study implies that overexpression of Oct4 might increase cell plasticity due to its powerful reprogramming potential while the optimization of the culture condition should be combined to complete the lineage specification.

The TF-induced reprogramming strategy is also applicable to ESCs and iPSCs to enhance their conversion efficiency into HPSCs. Since these cells are ready to differentiate, they tend to require fewer TFs for HSPC differentiation compared to mature somatic cells. The homeodomain TF HoxB4 and its related regulator Cdx4 are known to promote the hemogenic induction in PSCs both *in vitro* and *in vivo* [52, 53]. Interestingly, the differentiation potential of PSC-derived HSPCs can be directed by TFs. It is reported that the Gata2 and Etv2 combination stimulated ESCs to generate pan-myeloid progenitors, while replacement of Etv2 with Tal1 led to a skewed differentiation process towards the erythromegakaryocytic lineage instead [54]. Therefore, defining the role of each hematopoietic TF must be preceded to establish a reliable and efficient hematopoietic reprogramming strategy.

## 4. Utilization of Circulating HSPCs Directly Isolated from the Blood

Researchers have developed an *in vitro* RBC specification protocol from circulating HSPCs and proved the therapeutic potential of generated RBCs both in the preclinical and clinical settings [55–57]. Circulating HSPCs can be directly obtained from the bone marrow or blood collection. Due to the invasiveness of the bone marrow-harvesting procedure, PB and UCB are regarded as the most common sources for CD34^+^ HSPCs and *ex vivo*-produced RBCs. In addition, mononuclear cells isolated from buffy coats of blood donations could be differentiated into RBCs [58].

As a reservoir for CD34^+^ HSPCs, UCB has several advantages over PB [59, 60]. UCB-derived cells tend to have a relatively low chance of contamination with blood-borne infectious agents and aging-related cellular abnormalities such as spontaneous genetic mutations. UCB is known to contain a high frequency of immature, primitive progenitors with superior proliferative and differentiation potential. Indeed, Giarratana et al. have reported that UCB-derived HSPCs are capable of 140,000-fold expansion on average, while a proliferation plateau was only 16,500-fold and 29,000-fold for naturally collected and G-CSF-mobilized PB-HSPCs, respectively [56]. Furthermore, standardized UCB banking systems provide a reliable opportunity to utilize UCB with convenience. In contrast, PB supply is largely dependent on healthy volunteers, which makes it difficult to avoid ethical issues as well as technical problems to obtain clinical-grade CD34^+^ cells. These advantages of UCB lead to an increased application of UCB-derived cells compared to other human-originated cells both in basic and clinical research fields.

Considering the RBC count required for the conventional transfusion (2 × 10^12^ cells per single transfusion unit), the major obstacle to practical usage of blood-isolated HSPCs is their limited self-renewal capacity. Based on our observation, about 1~2 × 10^4^ CD34^+^ HSPCs can be isolated with 1 mL of UCB. Others have reported that ~10^9^ mononucleated cells are present in a single UCB unit (100~150 mL) while only 1-2% of which are CD34^+^ HSPCs [61]. Therefore, many attempts have been made to obtain sufficient number of HSPCs and/or erythroid lineage progenitors prior to induction of RBC maturation (Table 2).

### 4.1. Optimization of Conventional Culture Methods

Over the last few decades, several protocols have been developed to optimize the erythropoiesis-like process *in vitro*. Currently, most RBC generation methods use a serum free-liquid media culture system with various hematopoietic and erythropoietic factors [62]. It is noted that EPO mediates the expansion, survival, and differentiation of CFU-E and erythroid committed precursors while the genetic mutations in EPO or the EPO receptor gene lead to embryonic lethality with severe anemia [62, 63]. SCF is another essential factor for RBC development considering that disruption of SCF receptor kit signaling via mutation or antagonist treatment resulted in impaired erythropoiesis within the bone marrow due to a defect in BFU-E and CFU-E formation [64]. Although EPO and SCF combination has significantly increased the expansion of the CD34^+^ HSPC-derived CD71^+^ proerythroblast compared to EPO or SCF alone [65], they might have distinctive roles in RBC generation regarding the differential expression patterns of their receptors during erythroid lineage specification. Meanwhile, TPO, an important stimulant for the HSPC expansion, seems to induce megakaryocytic differentiation followed by a relative decline in RBC population [66], although it could stimulate the blood system recovery with enhanced erythroid progenitor expansion and RBC maturation as observed in a myelosuppressive mouse model [67]. Therefore, stage-specific factor combinations should be established to generate fully mature RBCs from blood-isolated HSPCs *in vitro* (Table 2).

In addition, coculture with stromal cells could not only stimulate the CD34^+^ HSPC proliferation but also improve the efficiency of RBC maturation. Giarratana et al. have demonstrated that the murine stromal cell MS-5 could successfully support the exponential expansion of UCB-isolated HSPCs and their maturation into functional, enucleated RBCs both *in vitro* and *in vivo* [56, 68]. Fujimi et al. have reported an advanced erythropoietic coculture condition using two different supporting cells in a stage-dependent manner [69]. In this study, PB-derived CD34^+^ cells were cocultured with telomerase gene-transduced human stromal cells during the so-called “first phase” to expand the HSPC population. These cells were forced to differentiate into erythroblasts using a conventional liquid culture method (second phase) then cocultured with macrophages from the third to the fourth phase for further expansion and maturation of RBCs. Using this method, researchers achieved an approximately 10^6^-fold expansion of the erythroblast from a single CD34^+^ cell. Moreover, the final phenotypical analysis on culture day 38 revealed that the coculture with a macrophage could increase the enucleated RBC proportion in the total cells by 40-70% to over 99%. Thus, theoretically, about 3 transfusion RBC units (6 × 10^12^ RBCs) can be generated from only 5‐6 × 10^6^ of PB-isolated CD34^+^ cells. Considering the higher proliferative potency of UCB-derived HSPCs than those of PB cells [56], it might be worthy to evaluate the yield of RBC generation from UCB-isolated HSPCs using this technique. Another report by Baek et al. has suggested the importance of stromal feeder cell origins during RBC generation [70]. They isolated CD34^+^cells from UCB then induced RBC differentiation for three weeks including the coculture step with either BM-MSCs or UCB-MSCs from days 8 to 21. Interestingly, the most efficient outcome was observed when the RBC specification process was supported by UCB-MSCs in which the final expansion fold was almost doubled compared to BM-MSCs. Although the underlying mechanism has not been elucidated, this study would provide a novel insight to apply UCB-derived cells for the RBC generation as it could provide the feeder MSCs as well as CD34^+^ HSPC-derived erythroid progenitors.

### 4.2. Application of the Bioreactor System or Biomimetic Niche

Hematopoiesis occurs in the bone marrow niche, and this specialized microenvironment consists of various cell types such as MSCs, osteoblasts, adipocytes, and endothelial cells with a well-organized extracellular matrix [71, 72]. They produce bioactive signaling compounds and provide the optimal mechanical forces through the complicated interactions. Moreover, the magnitude of cytokine expression levels and the oxygen concentration in the bone marrow niche are tightly regulated to maintain the optimal microenvironment for hematopoiesis. Indeed, HSPC expansion was greatly increased within the bone-derived scaffold containing BM-MSCs [73–75]. In this respect, it would not be surprising that many attempts have been made to recapitulate the hematopoietic niche *in vitro* with biomimetic materials. To generate an artificial niche, an adequate scaffold structure should be prepared first and then niche-supporting cells are introduced followed by HSPC seeding. MSCs derived from BM and UCB are the most frequently used niche-mimicking cell types since they possess both osteogenic and adipogenic potentials. In addition, several biocomparative materials have been pioneered to generate a niche-recapitulating structure for efficient blood production. In comparison with the conventional 2D culture system, a 3D scaffold with macroporous structure such as a fabricated polymer matrix made of either natural substance (e.g., collagen and fibronectin) or synthetic materials (e.g., L-lactic-co-glycolic acid and porous polyethylene glycol diacrylate) seems to be advantageous to maintain the highly potent, functional HSPCs for a long period of time [76–78]. One critical limitation of this method is that cells should be isolated from the niche-like structures for further lineage specification and maturation into RBCs. Thus, a biomimetic niche-dependent culture system needs technical modifications to directly obtain mature RBCs.

Recently, the bioreactor system also has been applied for the large-scale in vitro cell generation [79]. Since the culture media within the bioreactor are automatically replaced to maintain the optimized culture conditions, it is possible to obtain the large number of target cells beyond the laboratory level. In addition, the feeder-free system enables manufacturers to establish a xeno-free, cost-effective culture protocol, which will provide a great advantage for clinical applications. According to the most recent report, the authors demonstrated that theoretically, over 500 transfusable RBC units can be generated from only five million CB-derived CD34^+^ cells using the bioreactor method [80]; however, either the final enucleation rate or the total number of mature RBCs after filtration to remove nucleated cells was not reported. Therefore, the reproducibility and feasibility of these results should be confirmed prior to practical utilization.

### 4.3. Genetic Modifications for Immortalization of Blood-Derived Erythroblasts

Since HSPCs and committed erythropoietic progenitors isolated from UCB or PB gradually stop to proliferate within 2-3 weeks of culture and start to differentiate into mature RBCs instead, cell cycle regulators and pluripotency-inducing factors are common candidates for the genetic manipulation to maintain cell division as well as other immortalization strategies [81, 82]. It has been noted that the timing of the genetic modification is critical to obtain highly proliferative functional erythroblasts.

Cheng's group introduced iPSC-inducing factors Oct4, Sox2, Klf4, and c-Myc in combination with p53-siRNA to UCB-derived CD235^+^ erythroblasts. They found that Klf4, Sox2, and c-Myc overexpressed erythroblasts (iE cells) showed an extensive proliferation capacity (~10^68^-fold in a year) with the maintenance of their immature phenotype, while Oct4 expression was dispensable and even played a somewhat negative role on the long-term culture [83]. They further noted that only Sox2 and c-Myc could support the generation of immortalized RBC precursors. A comprehensive comparison of global transcriptome between iE cells and UCB-derived primary erythroblasts has revealed that several Sox2 families (Sox2, Sox4, Sox6, and Sox21) and Myb were upregulated in iE cells compared to primary cells, not only providing information about underlying mechanisms but also suggesting novel TF candidates for immortalized RBC progenitor generation. In another study, Geiler et al. focused on the potential role of the SPI-1 transcript PU.1, a key hematopoietic transcription factor, in erythropoiesis and hypothesized that overexpression of PU.1 might impede the RBC maturation and stimulate the RBC precursor expansion instead [84]. To prove the hypothesis, SPI-1 containing PiggyBac vector was transfected to PB-HSPC-derived erythroblasts. They observed the continuous expansion of transfected erythroblasts over 45 days without further maturation into RBCs based on their CD marker expression patterns (CD71^+^ CD117^+^) assessed by flow cytometry. Trakarnsanga et al. have demonstrated another immortalization protocol for erythroid progenitors obtained from PB-derived CD34^+^ cells [85]. In this report, PB-HSPCs were transduced with a Tet-inducible E6/E7 expression system then cultured in erythroid specification condition, to generate the immortalized adult erythroid precursors (referred to as BEL-A cells). They emphasized that BEL-A cells share several aspects of morphological and molecular characteristics with adult erythroblasts and reticulocytes including the expression patterns of membrane antigens and hemoglobin. BEL-A-derived RBCs after the enucleation process could transport oxygen in a similar manner to normal adult RBCs. Finally, the survival and maturation of BEL-A reticulocytes were confirmed in the murine circulation, implying their great potential for clinical uses. Taken together, these studies suggest that genetic engineering with reprogramming factors or hematopoietic regulators could convert primary erythroblasts into an immortalized cell line, which potentiates the feasibility of the *ex vivo* RBC product in the clinical field.

## 5. Current Limitations and Future Perspectives of RBC Generation

To establish a reliable, efficient RBC generation protocol, the best cell source for RBC induction must be determined first. The most prominent characteristics of ESCs and iPSCs are their unlimited expansion capacity. In particular, iPSCs might be a more favorable cell source than ESCs because they can be applied to the patient- or recipient-specific blood generation with additional gene modifications as described above [37, 38]. It is also possible to proceed through the whole protocol from the iPSC generation to RBC maturation under good manufacturing practice (GMP) conditions, which will be an essential requirement to use *in vitro*/*ex vivo*-generated biomaterials in the clinical field. On the other hand, general safety issues including the potential tumorigenic capacity of undifferentiated iPSCs should be resolved before the practical application of iPSC-derived products. Meanwhile, UCB contains primitive, multipotent HSPCs which can be easily differentiated into lineage-specific progeny with defined culture conditions. UCB also can provide other cellular components for hematopoietic culture maintenance such as MSCs and other hematopoietic lineage cells, which can support proliferation as well as differentiation of HSPCs. Finally, only UCB that meet the quantity (cell number) and safety criteria (free of any infectious agents) will be preserved in the banking system. Therefore, it is convenient to obtain quality-proven hematopoietic cells from UCB compared to other human-originated sources.

To translate *in vitro*/*ex vivo*-produced RBCs into the clinical field, several challenging issues should be overcome. First, phenotypic characterization of manufactured RBCs compared to naïve RBCs is necessary. Since PSCs and UCB-derived cells are closer to embryos than to adults in terms of developmental status, PSC- and UCB-derived RBCs tend to possess insufficient maturation patterns [28, 86]. Several studies have reported that RBCs derived from PSCs mainly express embryonic and fetal hemoglobin with little expression of adult hemoglobin [86, 87]. Similarly, UCB-derived RBCs contain a more immature type of globins than PB-derived ones, although insufficient RBC maturation could be completed *in vivo* [88, 89]. These immature hemoglobins are still functional and have even higher oxygen-binding affinity than adult ones [90]; however, intensive comparison between adult and fetal globins would be needed to determine the comparability of PSC- or UCB-derived RBCs. In addition, it has been noted that some of RBC surface antigens derived from UCB seem to be differentially expressed compared to those of naïve- or PB-derived RBCs [58]. Considering that surface antigen profiling is important to estimate the maturity of RBCs as well as to predict the safety and efficacy issues after transfusion, differences in the expression pattern of blood group antigens between normal and manufactured RBCs should be evaluated prior to clinical application.

Another major challenge is to produce a substantial number of RBCs for transfusion. It is noted that the dynamic suspension culture system with a bioreactor facility is regarded as a great substitute for the conventional 2D culture. Theoretically, 560 transfusion units, the highest yield ever, can be generated from one unit of UCB under the bioreactor system when cells were supplemented with SCF, EPO, and IL-3 [80]. In addition, researchers have successfully generated the immortalized erythroid progenitor line that has exponential growth potential and therefore can be a long-term source for *ex vivo* RBCs [83]. Either technique can be applied by itself; however, an optimized combination of these two strategies would produce a greater outcome. Of note, the efficient generation of RBCs also contributes to conducting various attempts with an advanced concept aimed at manufacturing utilizable cell products. Recently, Hawksworth et al. have demonstrated a proof of concept to improve RBC compatibility using a gene editing technique [91]. In this study, the 5 major blood group antigens including KEL, RHAG, ACKR1, FUT1, and GYPB were ablated from the immortalized erythroid cell line BEL-A via combinational gene targeting with the CRISPR-Cas9 system to minimize safety issues related to incompatible transfusion. The authors successfully generated multiple knockout erythroid lines without any off-target mutations and confirmed the ablation of each antigen expression using an indirect antiglobulin assay. Importantly, no noticeable physiological change during the differentiation and maturation process was observed in knockout cells compared to normal RBCs, suggesting that not only conventional but also the customized transfusion unit for recipients with rare blood types can be manufactured in a large scale from immortalized RBC lines. Overall, these continuous efforts to establish advanced strategies for a cost-effective, highly potent RBC culture system combined with engineering techniques would ultimately contribute to the practical utilization of *ex vivo*-generated RBCs in the near future.

## Figures and Tables

**Table 1:** Current cell sources and strategies for ex vivo RBC generation.

Cell source	PSCs		Circulating cells		Immortalized RBC lines
	ESCs	iPSCs	UCB	PB	
Strategy	PSCs are differentiated into hematopoietic lineage to generate RBCs		CD34^+^ HSPCs are isolated from the blood		HSPCs or erythroid progenitors are immortalized

Pros	(i) Superior expansion potential	(i) Superior expansion potential	(i) Direct source for HSPCs	(i) Direct source for HSPCs	(i) Theoretically unlimited expansion potential
	(ii) Established quality control criteria for GMP grade	(ii) Suitable for donor-specific transfusion	(ii) Established quality control criteria for GMP grade	(ii) RBCs from PB have more mature, adult-like phenotypes	(ii) Applicable for further gene editing
			(iii) Contains primitive HSPCs than PB		

Cons	(i) Low RBC induction efficacy	(i) Low RBC induction efficacy	(i) Limited expansion potential	(i) Limited expansion potential	(i) Safety criteria are needed
	(ii) Immature phenotype of generated RBCs	(ii) Immature phenotype of generated RBCs	(ii) Immature phenotype of generated RBCs	(ii) Contains a low number of HSPCs than UCB	
	(iii) Ethical and safety criteria are needed	(iii) Safety criteria are needed		(iii) Quality of HSPCs varies depending on individuals	

PSC: pluripotent stem cell; ESC: embryonic stem cell; iPSC: induced pluripotent stem cell; UCB: umbilical cord blood; PB: peripheral blood; HSPC: hematopoietic stem/progenitor cell.

**Table 2:** Current representative RBC-producing strategies using circulating HSPCs.

Culture methods			Total cell fold expansion^∗^	RBC maturation characteristics			Estimated transfusion unit^∗∗∗^	Reference
Strategy	Media	Period		Enucleation rate	Hb amount^∗∗^	HbA/HbF ratio		
3-step protocol	EIS (step 1)	18 days	~6 × 10^4^	81%	N.A	88 : 12	N.A	[68]
	ES (step 2)							
	E (step 3)							

3-step protocol	FST (step 1)	21 days	6‐10 × 10^5^	4%	40–50 g of Hb	40 : 60	1-1.5 units	[89]
	EIS (step 2)							
	IE (step 3)							

4-step protocol with feeder cells	FST+feeder (step 1)	38 days	4.35 × 10^6^	99.4%	~30 g of Hb	45 : 55	8.8 units	[69]
	EIS (step 2)							
	E+feeder (step 3-step 4)							

2-step protocol with feeder cells	EIS (step 1)	21 days	8.032 × 10^4^	64%	19 g of Hb	N.A	0.75 units	[70]
	EIS+feeder (step 2)							

2-step protocol with bioreactor	EIS (step 1)	21 days	2.25 × 10^8^	N.A	30.8 g of Hb	50.8 ± 10 : 47.3 ± 7	560 units	[80]
	EI (step 2)							

E: erythropoietin; F: fms-related tyrosine kinase 3 ligand; GM: granulocyte-macrophage colony-stimulating factor; I: interleukin 3; S: stem cell factor; T: thrombopoietin. ^∗^Fold expansion of CD34^+^ HSPCs and erythroblasts; ^∗∗^hemoglobin amount of 10^6^ CD34^+^ cell-derived RBC; ^∗∗∗^referred to the authors' evaluation. 1 unit of RBC transfusion is approximately 2 × 10^12^ RBCs.
